# Cor Triatriatum Dexter: An Innocent Bystander

**DOI:** 10.14797/mdcvj.1082

**Published:** 2022-04-18

**Authors:** Stephanie C. Fuentes Rojas, Gerald Lawrie, Nadeen N. Faza

**Affiliations:** 1 Houston Methodist DeBakey Heart & Vascular Center, Houston, Texas, US

**Keywords:** cor triatriatum dexter, multimodality imaging

## Abstract

Cor triatriatum dexter is a rare congenital heart defect with a varied clinical presentation ranging from asymptomatic to right heart failure. Accurate diagnosis is imperative as it may affect clinical decision making. We present a multimodality imaging assessment of cor triatriatum dexter in a 70-year-old woman with severe tricuspid regurgitation.

A 70-year-old woman with persistent atrial fibrillation and sick sinus syndrome status post dual-chamber pacemaker presented with dyspnea on exertion and lower extremity edema.

A transthoracic echocardiogram (TTE) showed severe tricuspid regurgitation (TR) with severely enlarged right atrium (RA) and right ventricle (RV). RV systolic function was mildly decreased, but left ventricular function was normal. The TTE also revealed a membranous structure that appeared to be attached to the interatrial septum. Transesophageal echocardiogram (TEE) was done to evaluate the membrane and suitability of transcatheter options for TR; it showed torrential TR due to annular dilation with a coaptation gap of 1.7 cm and no evidence of pacemaker lead impingement. A continuous membrane traversing through the RA and attaching to the intra-atrial septum (IAS) was noted. Color Doppler showed flow across the membrane. Cardiac magnetic resonance imaging, which was done to assess RV function and further characterize this membrane, showed that the membrane effectively divided the RA into two chambers and attached to the IAS. This was consistent with cor triatriatum dexter. Coronary computed tomography angiography (CCTA), which was ordered for coronary evaluation, confirmed the presence of cor triatriatum dexter (***Figure 1, Video 1***). After multidisciplinary review of the multimodality imaging, the decision was made to proceed with surgical tricuspid valve repair given the wide coaptation gap and the presence of cor triatriatum dexter, which would complicate catheter maneuvering in the RA during a transcatheter intervention. Intraoperative inspection of the cor triatriatum dexter did not reveal obstruction or hemodynamic significance, so it was not resected. The patient underwent successful full ring annuloplasty with a 31-mm St Jude Attune ring and 3-mm adjustment to the posterior leaflet segment.

**Figure 1:**
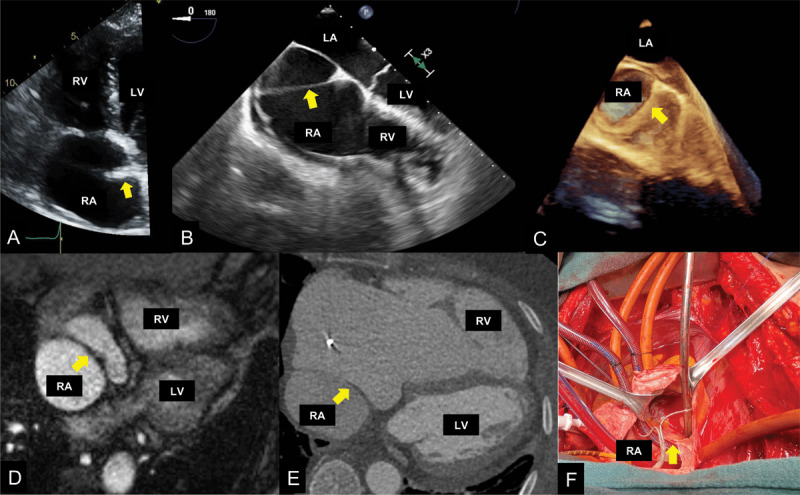
**(A)** Transesophageal echocardiogram (TEE) depicts a noncontinuous linear structure that seems to attach to the intra-atrial septum. **(B)** TEE further defines the membrane as a continuous structure, with color Doppler showing flow across membrane (see Video 1). (**C)** 3-dimensional (3D) TEE confirms what was seen in 2D imaging. **(D)** Cardiac magnetic resonance imaging confirms cor triatriatum dexter. **(E)** Coronary computed tomography angiography depicts low-attenuation structure traversing through right atrium. **(F)** Intraoperatively, cor triatriatum dexter was confirmed, but importantly, right ventricular inflow obstruction was excluded. RV: right ventricle; LV: left ventricle; LA: left atrium; RA: right atrium

**Video 1:** Transesophageal echocardiogram depicting continuous membrane. Color Doppler shows flow across this membrane. This also can be viewed at *https://youtu.be/wfF14wMF4C4*.

In cor triatriatum dexter, the right valve of the sinus venosus fails to regress, thus separating the trabeculated and smooth portions of the right atrium into two separate chambers.^1^ This leads to a triatrial heart (cor triatriatum). The clinical significance is varied and depends on the degree of chamber separation. With mild separation, patients may be asymptomatic; however, with more severe separation, they may present with right heart failure because the separation may create obstruction of flow into the RV.^2^ In neonates with atrial septal defects, cor triatriatum may lead to cyanosis due to right-to-left shunt.^3^ As such, its diagnosis in the correct clinical context is important. In our patient, the recognition of cor triatriatum played a key role in the multidisciplinary discussion of a transcatheter versus surgical approach to tricuspid regurgitation treatment.
